# Bird‐related non‐fibrotic hypersensitivity pneumonitis with bronchoalveolar lavage fluid eosinophilia that developed after COVID‐19 vaccination: A case report

**DOI:** 10.1002/rcr2.1294

**Published:** 2024-02-07

**Authors:** Takuma Katano, Tomoyuki Ogisu, Akira Satou, Toshiyuki Yonezawa, Hiroyuki Tanaka, Satoru Ito

**Affiliations:** ^1^ Department of Respiratory Medicine and Allergology Aichi Medical University Nagakute Japan; ^2^ Department of Surgical Pathology Aichi Medical University Nagakute Japan

**Keywords:** bronchoalveolar lavage fluid, COVID‐19, eosinophilic pneumonia, hypersensitivity pneumonitis

## Abstract

A 60‐year‐old man who had been keeping seven budgerigars and four cockatiels in his house for 2 years developed dyspnea and was admitted to our hospital the day after receiving the second dose of the messenger RNA coronavirus disease 2019 vaccination. Chest high resolution computed tomography (HRCT) showed bilateral ground glass opacities without nodules or mosaic attenuation. IgG specific for budgerigars was positive. Although his respiratory symptoms were resolved without corticosteroid therapy, he developed severe dyspnea soon after the discharge to his home. The results of bronchial alveolar lavage fluid obtained at the initial admission and after the provocation challenge showed elevation of lymphocytes (34%) and eosinophils (37%). We finally diagnosed him with non‐fibrotic bird‐related hypersensitivity pneumonitis. His condition and HRCT findings were improved by corticosteroid treatment. All his birds were given away. He has not experienced any recurrence or deterioration of respiratory function even after withdrawal of corticosteroid.

## Graphical abstract

Hypersensitivity pneumonitis (HP) is caused by exposure to an overt or occult inhaled antigens in susceptible individuals. Bird‐related HP is now one of the major causes of HP. Several cases of eosinophilic pneumonia (EP) associated with the COVID‐19 vaccination have been reported. Here, we report a case of non‐fibrotic, bird‐related HP with bronchoalveolar lavage fluid (BALF) eosinophilia equivalent to EP after COVID‐19 vaccination.

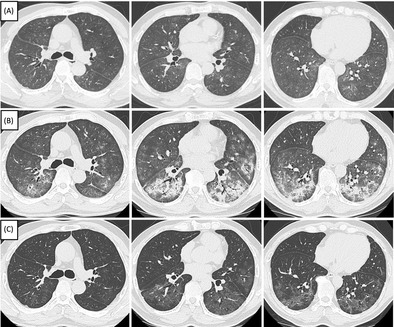

## INTRODUCTION

Hypersensitivity pneumonitis (HP) is caused by exposure to an overt or occult inhaled antigens in susceptible individuals.^1^ HP is classified into two types: non‐fibrotic HP (an acute and predominantly inflammatory type) and fibrotic HP (a chronic and predominantly fibrotic type).^1^ Bird‐related HP is now one of the major causes of HP. In Japan, most bird‐related HP case are fibrotic type and the non‐fibrotic type is less common.

As an effective countermeasure against the coronavirus disease 2019 (COVID‐19) pandemic, messenger RNA vaccinations have been administered around the world. Several cases of eosinophilic pneumonia (EP) associated with the COVID‐19 vaccination have been reported.^2^ We report a case of non‐fibrotic, bird‐related HP with bronchoalveolar lavage fluid (BALF) eosinophilia equivalent to EP after COVID‐19 vaccination.

## CASE REPORT

**FIGURE 1:**
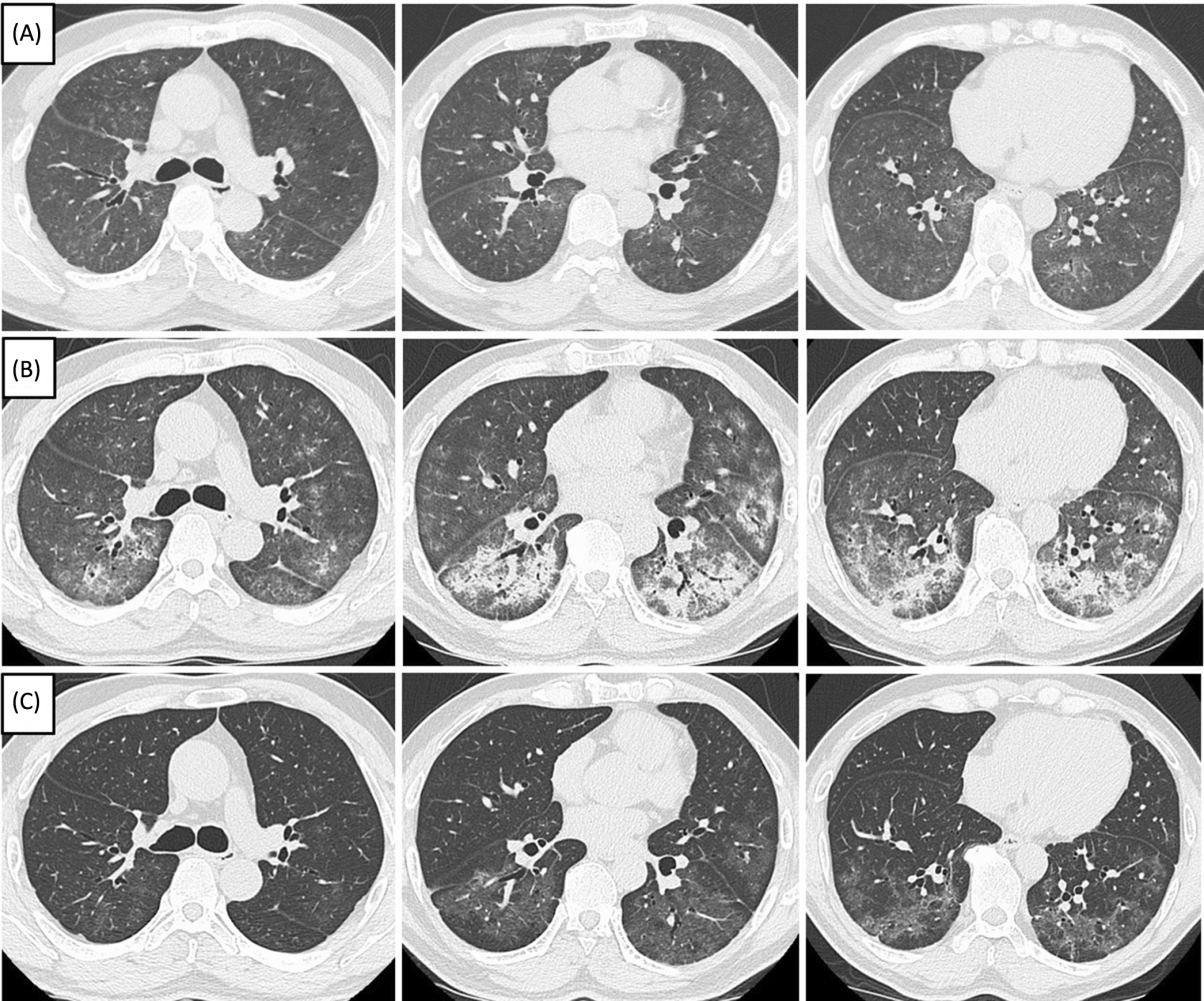
Images of chest CT at the first admission (A), at the readmission (B), and 2 weeks after starting the corticosteroid treatment (C).

A 60‐year‐old man was referred to emergency room of Aichi Medical University Hospital from an outpatient clinic due to dyspnea the day after administration of the second dose of the BNT162b2 messenger RNA COVID‐19 vaccine (Pfizer/BioNTech). He had no previous history of respiratory or allergic diseases. He was a current smoker (40 pack‐years) with no history of changes in cigarette brand. He had been keeping seven budgerigars (*Melopsittacus undulatus*) and four cockatiels (*Nymphicus hollandicus*) indoors for two years. He was admitted to our hospital due to suspected pneumonia and treatment with sulbactam ampicillin was initiated. His body temperature was 36.3°C and his partial pressure of arterial oxygen was 68.7 Torr with 2 L/min oxygen inhalation via nasal cannula. Chest high‐resolution computed tomography (HRCT) showed bilateral ground glass opacities without a nodule or mosaic attenuation (Figure 1A). Laboratory data showed that peripheral white blood cell and eosinophils counts were within normal range, and levels of serum LDH, C‐reactive protein (CRP), Kreb von den Lugen‐6, and SP‐D were high (Table 1). The results of a pulmonary function test revealed restrictive change with decreased diffusing capacity (Table 1). The following diagnostic possibilities were considered: interstitial lung disease (ILD) such as collagen tissue disease associated ILD, drug‐induced ILD and HP. Two days after admission, BAL and transbronchial lung biopsies (TBLB) were performed using a flexible bronchoscope. The results of BALF obtained from the B5 right middle lobe (46% of recovery rate) showed elevation of total cell counts (5.27 × 10^5^/mL), eosinophils (37%), and lymphocytes (34%) with a low CD4/CD8 ratio (0.37). No bacterium or fungus was isolated from cultures of BALF. A TBLB specimen obtained from the B8 right lower lobe showed lymphocytic alveolitis without granuloma and eosinophilic infiltration. The serum immunoglobulin (Ig) E level was low, and IgE specific for feathers and droppings of budgerigars were negative (Table 1). Moreover, tests for autoimmune antibodies were negative (Table 1). His respiratory symptoms improved 4 days after the admission without corticosteroid treatment. Six days after the admission, his peripheral artery oxygen saturation was 96% without supplemental oxygen, and levels of serum LDH (256 U/L) and CRP (0.3 mg/dL) were decreased. Moreover, IgG specific for budgerigars proved to be positive. Therefore, bird‐related HP was strongly suspected. Twelve days after the admission, we discharged him to his home as a provocation challenge after he gave informed consent.

**TABLE 1:** Laboratory findings and results of pulmonary function test on admission.

Haematology		Immunochemistry		Arterial blood gas	O_2_ 2 L/min
WBC	7500/μL	KL‐6	1726 U/mL	pH	7.488
Neu	67.7%	SP‐A	71.6 ng/mL	PO_2_	68.7 Torr
Lym	23.4%	SP‐D	1630 ng/mL	PCO_2_	29.5 Torr
Mon	6.6%	CH50	56.5 mg/dL	HCO^3−^	20.0 mmoL/L
Eos	1.6%	ESR	5 mm/h		
RBC	4.95 × 10^3^/μL	IgG	943 mg/dL	Pulmonary function test	
Hb	16.5 g/dL	IgA	195 mg/dL	VC	3.17 L
Plt	237 × 10^3^/μL	IgM	63 mg/dL	%VC	74.1%
		IgE	60 IU/mL	FVC	3.17 L
Biochemistry		Budgerigars feather IgE Ab	<0.10 UA/mL	%FVC	76.0%
TP	6.5 g/dL	Budgerigars dropping IgE Ab	<0.10 UA/mL	FEV_1_	2.55 L
Alb	3.9 g/dL	Budgerigars IgG Ab (Cut‐off value)	10.3 mgA/L (<8.7 mgA/L)	%FEV_1_	73.3%
BUN	14.7 mg/dL	Trichosporon asahii IgG Ab	0.01 CAI	FEV_1_/FVC	0.80
Cre	0.98 mg/dL	Antinuclear Ab	<×40	DLco	10.05 mL/min/mmHg
Na	141 mmol/L	Anti ARS Ab	<1.0 U/mL	%DLco	47.6%
K	4 mmol/L	Anti SS‐A/Ro Ab	<1.0 U/mL		
LDH	327 U/L	Anti CCP Ab	<0.6 U/mL		
AST	29 U/L	Rheumatoid factor	<3.0 IU/mL		
ALT	29 U/L	MPO‐ANCA	<1.0 U/mL		
CRP	0.87 mg/dL	PR3‐ANCA	<1.0 U/mL		

Abbreviations: Ab, antibody; ARS, aminoacyl tRNA synthetase; CAI, corrected absorbance index; CCP, cyclic citrullinated peptides; CH50, 50% hemolytic complement activity; DLco, diffusing capacity of the lungs for carbon monoxide; ESR, erythrocyte sedimentation rate; FEV1, forced expiratory volume in 1 s; FVC, forced vital capacity; Ig, immunoglobulin; KL‐6, Kreb von den Lugen‐6; PR3‐ANCA, proteinase3‐anti‐neutrophil cytoplasmic antibody; MPO‐ANCA, myeloperoxidase‐anti‐neutrophil cytoplasmic antibody; VC, vital capacity.

**FIGURE 2:**
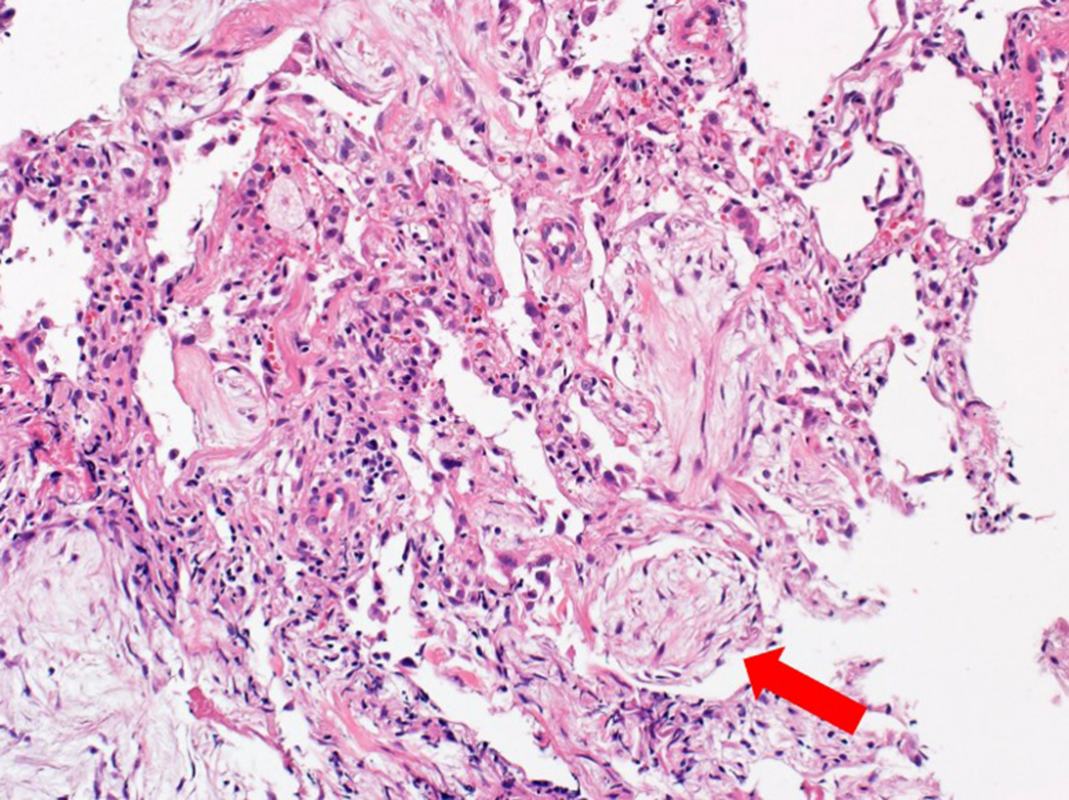
A lung specimen obtained by transbronchial lung biopsy showed a Masson body (red arrow), intraluminal plugs of granulation tissue, and lymphocytic infiltration. An original magnification view (×200).

Soon after returning home, he developed dyspnea, and he was readmitted to our hospital 5 days after the discharge. His peripheral artery oxygen saturation was 90% without supplemental oxygen, and laboratory data at readmission showed elevation of levels of serum LDH (346 U/L), CRP (1.64 mg/dL), Kreb von den Lugen‐6 (2031 U/mL). Since his respiratory symptoms and radiological findings (Figure 1B) were worsened 10 days after the readmission, we performed BAL and TBLB again. The results of the second BALF (64% of recovery rate) showed elevation of total cell counts (8.37 × 105/mL), lymphocytes (34%) and eosinophils (37%), similar to findings in the first examination. Histopathology of TBLB showed intraluminal plugs of granulation tissue (Masson bodies) (Figure 2), in addition to the same findings as the first examination. Finally, he was diagnosed with non‐fibrotic bird‐related HP.

He received intravenous methylprednisolone (m‐PSL) (1000 mg per day) for 3 days followed by PSL 60 mg per day orally for respiratory failure. His condition and HRCT findings (Figure 1C) were improved 2 weeks after starting the corticosteroid treatment. After all his birds were given away and his house was cleaned, he was discharged home with PSL 20 mg per day orally on the 27th day after the readmission. The dose of PSL was gradually tapered and finished 5 months after the readmission. He has not experienced any recurrence or deterioration of respiratory function for 2 years after the second discharge.

## DISCUSSION

In this case, non‐fibrotic bird‐related HP developed after the second dose of a COVID‐19 vaccination. BALF results showed increases of both lymphocytes and eosinophils. To our knowledge, this is the first report of bird‐related HP with BALF eosinophilia equivalent to EP.

The following three domains are important for the diagnosis of HP: (1) exposure identification, (2) CT radiological patterns and (3) BALF lymphocytosis/histopathological findings.^1^ In this case, it is difficult to definitively diagnose HP based on imaging patterns, and BALF and pathological findings. In addition to specific IgG antibodies, the worsening of respiratory symptoms, and laboratory and imaging findings due to provocation challenge led to the diagnosis of HP.

The patient's innate immune response, the intensity of the avian bio‐aerosol, and the degree and duration of exposure are important in the risk of developing bird‐related HP.^3^ In this case, there was no change in the intensity or degree of exposure to the avian protein antigen prior to onset. The immune response to the BNT162b2 vaccination was strikingly increased following the second vaccination. In our case, the COVID‐19 vaccination possibly activated his immune response and contributed to the development of HP.

The diagnostic criteria for acute EP, the modified Philit criteria, include BALF eosinophilia greater than 25%. Interestingly, our case showed BALF eosinophilia (37%) equivalent to EP. Caillaud et al. reported that BALF in 139 cases of HP revealed mean percentages of BALF lymphocytes and eosinophils of 53.2% and 1.2%.^4^ In contrast, there have been reports of BALF eosinophilia in bird‐related HP.^5^ However, there was no previous report showing such marked BALF eosinophilia as in our case. We experienced a rare case of bird‐related HP in which COVID‐19 vaccination may have contributed to BALF eosinophilia and HP development.

## AUTHOR CONTRIBUTIONS

Takuma Katano, Tomoyuki Ogisu and Satoru Ito conceived and designed the work. Takuma Katano and Satoru Ito drafted the manuscript. Takuma Katano, Tomoyuki Ogisu, Toshiyuki Yonezawa and Hiroyuki Tanaka collected clinical data. Akira Satou examined pathology. All authors have read and approved the final version of the manuscript.

## CONFLICT OF INTEREST STATEMENT

None declared.

## ETHICS STATEMENT

The authors declare that appropriate written informed consent was obtained for the publication of this manuscript and accompanying images.

## Data Availability

The data that support the findings of this study are available on request from the corresponding author. The data are not publicly available due to privacy or ethical restrictions.
